# Comparative proteomic analysis of the shoot apical meristem in maize between a ZmCCT-associated near-isogenic line and its recurrent parent

**DOI:** 10.1038/srep30641

**Published:** 2016-07-29

**Authors:** Liuji Wu, Xintao Wang, Shunxi Wang, Liancheng Wu, Lei Tian, Zhiqiang Tian, Ping Liu, Yanhui Chen

**Affiliations:** 1 Henan Agricultural University and Synergetic Innovation Center of Henan Grain Crops, Zhengzhou 450002, China; 2 Key Laboratory of Physiological Ecology and Genetic Improvement of Food Crops in Henan Province, Zhengzhou 450002, China; 3 Crop Designing Center, Henan Academy of Agricultural Science, Zhengzhou 450002, China

## Abstract

The *ZmCCT*, one of the most important genes affecting photoperiod response, delays flowering under long-day conditions in maize (*Zea mays*). In this study we used the isobaric tags for relative and absolute quantification (iTRAQ) technique-based proteomics approach to identify differentially expressed proteins between a near-isogenic line (NIL) and its recurrent parent, contrasting in alleles of *ZmCCT*. A total of 5,259 distinct proteins were identified. Among them, 386 proteins were differentially expressed between NIL-cml line (ZmCCT-positive) and H4 line (ZmCCT-negative). Functional categorization showed that the differentially proteins were mainly involved in energy production, photosynthesis, signal transduction, and cell organization and biogenesis. Our results showed that during shoot apical meristem (SAM) development cell division proteins, carbohydrate metabolism–related proteins, and flower inhibition-related proteins were more abundant in the ZmCCT-positive line than the ZmCCT-negative line. These results, taken together with morphological observations, showed that the effect of ZmCCT on flowering might be caused by its effect on one or all of these biological processes. Although the exact roles of these putative related proteins remain to be examined, our results obtained using the proteomics approach lead to a better understanding of the photoperiodicity mechanism in maize plants.

The photoperiod has been recognized as an important environmental signal required for the normal growth and breeding of higher plants since as early as 1920^1^. For example, in the model plant *Arabidopsis thaliana*, long days (LDs) induce flowering, whereas short days (SDs) inhibit the process. In contrast, SD plants, such as rice, can be induced to flower under SD conditions of fall^2^,^3^. In recent years, the molecular mechanisms mediated by the photoperiod have been studied in various plants^4^,^5^. A considerable number of the genetic and molecular components related to understanding the photoperiodic control of flowering time have also been characterized in *Arabidopsis*^6^,^7^.

In the case of maize, numerous quantitative trait loci (QTLs) affecting the flowering time have been identified. These QTLs are dependent on environmental conditions and a major QTL was mapped in the region of bin 10.04 of maize chromosome 10^8^,^9^,^10^. We recently reported the isolation of rice *Ghd7* gene homologs in maize, and a comprehensive analysis of *ZmCCT* using forward and reverse genetics showed that *ZmCCT* was a candidate *Ghd7* homolog and was associated with this major QTL^11^,^12^.

In the post-genome era, proteomics is becoming an important tool, which is used in different systems ranging from human disease to evaluating plants in response to virus pathogen or non-inductive conditions^13^,^14^,^15^. In the past decades, two-dimensional gel electrophoresis (2-DE) has been widely used to separate and analyze proteins of interest. However, 2-DE is often unable to analyze low-abundance proteins and basic/hydrophobic proteins, while mass spectrometry based proteomics technology enables simultaneous analysis and identification of proteins of low abundance, extreme pI values, and high molecular weights^16^,^17^,^18^,^19^,^20^.

In this work, we compare the changes in phenotype and protein abundance during the transition from vegetative growth to reproductive growth in a near-isogenic line (NIL) and its recurrent parent (the genetic context of the ZmCCT-containing or ZmCCT-deficient). With these measurements, a total of 386 proteins with markedly altered expression were identified which could be the direct or indirect targets of ZmCCT. This study will contribute to a better understanding of the molecular mechanism of photoperiodicity in maize.

## Results and Discussion

### Phenotypic differences between NIL-cml and H4 under LD conditions

In this study, the responses to photoperiod and morphology of two maize lines, NIL-cml and H4, were observed at different development stages. The plant height and flowering time of the two maize lines were significantly different under LD conditions. As seen in Table 1, NIL-cml showed significant delays in flowering and increased plant height compared with H4. Days to pollen shedding and days to silk of the NIL-cml (71.5 ± 0.26 days and 79.2 ± 0.43 days) were delayed by more than 7.2 and 9.7 days compared with the H4 (64.3 ± 0.32 days and 69.5 ± 0.22 days), respectively. These results indicated that H4 was considerably less sensitive to the LD photoperiod and NIL-cml was more sensitive to photoperiod.

**Figure 1:**
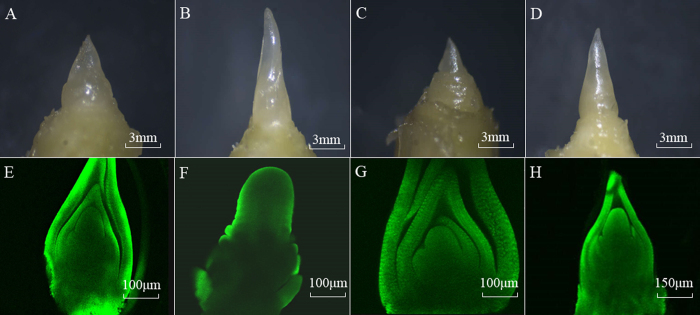
Changes in morphology of SAM from the NIL-cml and H4 lines (n = 10) under LD conditions. (**A,B**) are, respectively, the V3 and V6 of H4 SAM; (**C,D**) are, respectively, the V3 and V6 of NIL-cml SAM. (**E,F**) are, respectively, the V3 and V6 of H4 SAM was observed under Leica TCS-SP2 laser scanning microscope; (**G,H**) are, respectively, the V3 and V6 of NIL-cml SAM was observed under Leica TCS-SP2 laser scanning confocal microscope.

Under LD conditions, the SAMs were observed at the V3 and V6 stages of NIL-cml (NIL-cml-3 and NIL-cml-6) and H4 (H4-3 and H4-6). The morphology of the NIL-cml SAMs was observed from the V3 and V6 stage, and no visible morphological difference was observed between the V3 and the V6 stage. In contrast, the H4 SAM had already elongated and become a cone at the V6 stage (Figs 1 and S1). Thus, the NIL-cml showed extremely late flowering compared with the H4 under LD conditions.

Photoperiod signal is perceived in the leaf primordia, and then the signal is translocated to the SAM, where it stimulates the onset of flowering^21^. As seen in Fig. 1, the H4 line exhibited normal vegetative and reproductive growths. In contrast, the NIL-cml line showed significant delays in vegetative and reproductive growths under LD conditions. These results reinforce the role of *ZmCCT* as a core photoperiod component; floral initiation and development were found to be delayed by *ZmCCT* in LD conditions^12^,^22^.

### The expression levels of *ZmCCT* in NIL-cml and H4

In order to evaluate the temporal and spatial expression pattern of *ZmCCT*, we quantified the expression of *ZmCCT* mRNA levels using qPCR. During the V3–V10 stage, *ZmCCT* was consistently expressed in the NIL-cml SAMs, however, high levels of transcripts were detected during the V3 to V6 stage and then declined with the following development stages in NIL-cml (Fig. S2A). Low transcript level was detected at all developmental stages in H4. Figure S2B shows that *ZmCCT* was expressed at the highest levels in NIL-cml SAMs, while it was also detected at moderate levels in leaves and stems and at low levels in the root, low transcript level was detected at all tissues in H4. This result indicated that *ZmCCT* was expressed at higher levels in NIL-cml than in the H4 plant.

### iTRAQ analysis of potential proteins in NIL-cml and H4

For proteomic analysis, an experimental scheme was showed in Fig. S3. The SAMs were collected at the V3 and V6 stages from both lines. Each sample was collected from six different, randomly selected plants. Three biological replicates were collected at the same time. Then we compared the protein abundance ratios between the H4 and NIL-cml SAMs at the V3 and V6 stages (NIL-cml-3/H4-3, NIL-cml-6/H4-6, NIL-cml-6/NIL-cml-3 and H4-6/H4-3).

A comparative proteome survey was generated using the iTRAQ technique. A total of 23,767 high-quality unique peptides were obtained in this study. 5,259 distinct proteins were identified and quantified in three independent biological replicates. In addition to a 95% identification confidence level, a ratio of >1.5-fold change (up or down) was used to for further filter candidates^23^,^24^,^25^. In addition, to validate the reproducibility of their iTRAQ analyses among replicates, the Venn diagrams were showed in Fig. S4. And it indicated that about 90% shares a mostly similar coverage of the differentially expressed proteins (Fig. S4). Using these criteria, a total of 386 proteins (at least one unique peptides) were identified as being differentially expressed between the NIL-cml and H4 in these three replicates. Among them 154 proteins were identified with at least two unique peptides (Supplementary Tables S2 and S3).

### Protein ontology analysis and ZmCCT-associated protein identification

**Figure 2:**
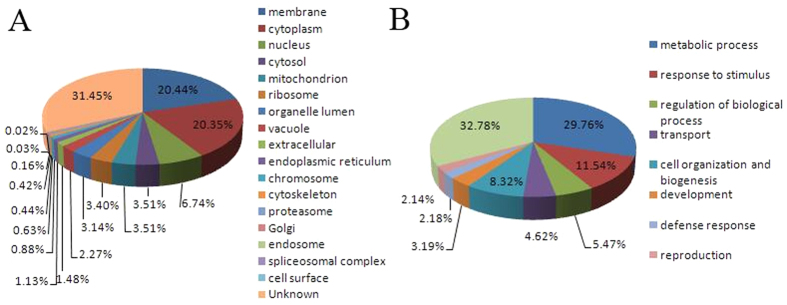
Functional classification and distribution of all 5,259 identified proteins. (**A**) Subcellular localization and (**B**) biological function/molecular function. Functions of the proteins were identified using the National Center for Biotechnology Information and UniProtKB databases. Detailed information on the proteins identified can be found in Supplementary Table S2.

For the subcellular localization analysis, the 5,259 identified proteins were assigned to 12 different cellular compartments, including primarily the nucleus (6.74%), membranes (22.44%), cytoplasm (20.35%), and mitochondria (3.51%); 30.45% proteins were with unknown subcellular localization information (Fig. 2A). Based on their biological process annotation, 29.76% of the proteins were found to be involved in metabolic processes, 11.54% in response to stimulus, and 5.47% in biological processes (Fig. 2B). Based on their putative molecular functions, the differentially expressed proteins were classified into seven groups: energy production, photosynthesis, signal transduction, stress and defense, carbohydrate metabolism, protein metabolism, and unknown function (Supplementary Table S3).

### Protein expression pattern in NIL-cml and H4 at V3 and V6 stages

**Figure 3:**
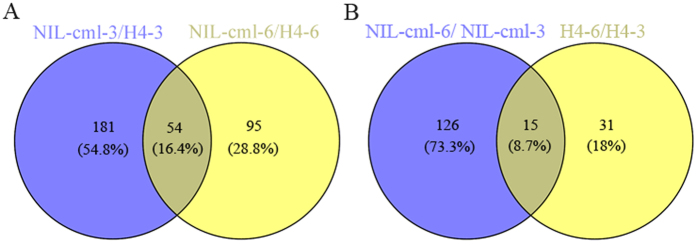
Comparative Analysis of differentially expressed proteins between NIL-cml and H4 in V3 and V6 stage. Venn diagram showing the distribution of common and exclusive proteins between NIL-cml-3/H4-3 and NIL-cml-6/H4-6 (**A**), NIL-cml-6/NIL-cml-3 and H4-6/H4-3 (**B**), respectively. The areas shown in the diagram are not proportional to the number of proteins in each group.

**Figure 4:**
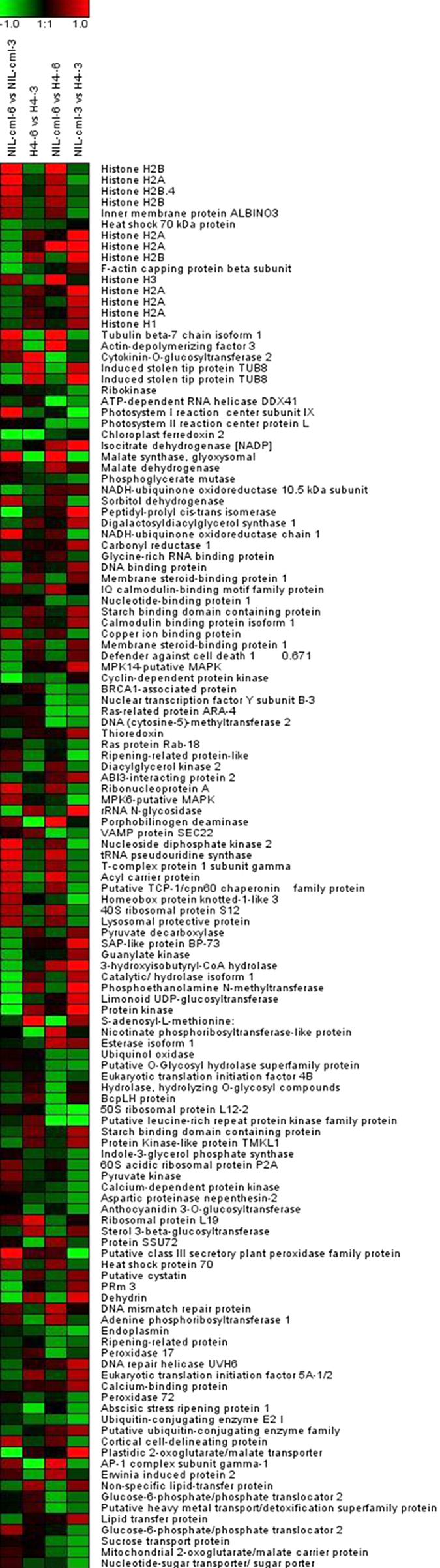
Analysis of changes in protein expression during vegetative to reproductive transition. Expression ratios of NIL-cml-3/H4-3, NIL-cml-6/H4-6, H4-6/H4-3, and NIL- cml-6/NIL- cml-3 were calculated based on a log2 scale. Red color indicates up-regulated proteins, and green indicates down-regulated proteins.

Of the 386 differentially expressed proteins, a total of 235 proteins showed differences between NIL-cml-3/H4-3 (V3 stage), including 113 proteins that were up-regulated and 122 were down-regulated in NIL-cml line. At the V6 stage, 149 proteins were significant up-(52) or down-(97) regulated in NIL-cml-6/H4-6 (Figs 3 and 4, Supplementary Tables S2 and S3). 172 differentially expressed proteins were identified between the V3 and V6 stages (NIL-cml-6/NIL-cml-3 and H4-6/H4-3). 141 showed differences in the ZmCCT-positive maize NILs (NIL-cml-6/NIL-cml-3). Of these, 46 proteins were up-regulated and 95 were down-regulated. 46 proteins showed differences in the ZmCCT-negative maize NIL (H4-6/H4-3). Of these, 15 were up-regulated and 31 were down-regulated (Fig. 4, Supplementary Tables S2 and S3).

Venn diagram analyses of the differentially regulated proteins were shown in Fig. S3. There were 21 proteins that showed changes in both NIL-cml-3/H4-3 and NIL-cml-6/H4-6; 8 proteins showed changes in NIL-cml-6/NIL-cml-3 and H4-6/H4-3.

**Figure 5:**
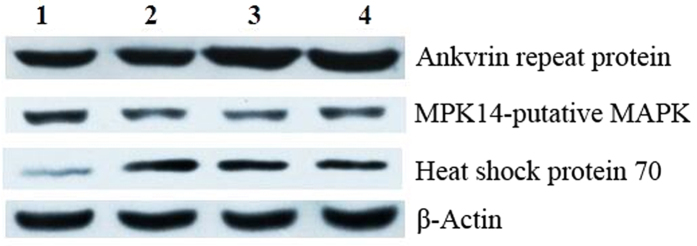
Western blot analysis of Ankyrin repeat protein, Heat shock protein 70, and MPK14-putative MAPK levels in NIL-cml (1: NIL-cml-3; 2: NIL-cml-6) and H4 (3: H4-3; 4: H4-6) samples. Expression level of β-actin was used as loading control. These experiments were repeated three times, with consistent results.

To validate the data from proteomics experiments, three proteins were randomly selected to verify the expression level via western blot analysis (Fig. 5). An Ankyrin repeat protein was significantly up-regulated in the H4 group when compared with NIL-cmlCK group (control). The expression of Heat shock protein 70 was elevated in NIL-CML-6 and H4-3 SAMs than that in NIL-CML-3 and H4-6 SAMs, independently. In contrast, the expression level of MPK14-putative MAPK was significantly down-regulated in the H4 group when compared with NIL-cmlCK group (control). The western blot results were consistent with the iTRAQ data, thus strongly supporting the reliability of the proteomics data.

### Proteins involved in cell organization and biogenesis

In plants, tubulin and actin are known to play key roles in cell proliferation, organ growth, and regulation of floral bud morphogenesis from the vegetative to the reproductive phase^26^,^27^,^28^,^29^. Our data showed that the expression levels of some tubulins and actins undergo significant changes during floral bud differentiation (V3 to V6 stage). These proteins showed a greater abundance in NIL-cml than in the H4 plants (NIL-cml-3/H4-3 and NIL-cml-6/H4-6); while the levels of these proteins are stable in the ZmCCT-negative maize line (H4-6/H4-3) from the vegetative to the induced stage (Supplementary Table S3), suggests that ZmCCT may have a large effect on stem growth by directly or indirectly inducing cytoskeletal proteins in the photoperiod-sensitive cultivar. This was consistent with the previous observation that *Ghd7* had marked effects on vascular development and stem growth^11^. Collectively, we speculate that the highly abundant accumulation of tubulin and actin in ZmCCT-positive plants might enhance cell division and growth during SAM development.

We also identified 12 histone proteins (Supplementary Table S3) whose expression level in ZmCCT-positive plants was higher than that in ZmCCT-negative plants (NIL-cml-3/H4-3 and NIL-cml-6/H4-6). This was consistent with other research in ZmCCT-positive maize NIL (NIL-cml-6/NIL-cml-3) suggesting that ZmCCT may induce histone protein in NIL-cml plants. In *Arabidopsis*, histone proteins greatly influence a wide range of developmental processes, including leaf development, repression of the transition to flowering, and potentiation of transcriptional activation^30^,^31^. During vegetative growth, histone H2A directly promotes FLOWERING LOCUS C (FLC) expression and thus leads to delayed flowering^32^. These results suggest that ZmCCT may induce histone protein and the expression of ZmFLC rises subsequently, thereby repressing the transition from the vegetative state to the reproductive state in maize.

### Proteins involved in carbohydrate and energy metabolism

Plants need a large quantity of ATP for sufficient energy for growth, flower development, reproduction, and stress responses^33^,^34^. In this proteomic study, 20 proteins involved in energy metabolism, photosynthesis, and carbohydrate metabolism were differentially regulated under LD conditions, providing evidence that ZmCCT can affect key metabolic processes (e.g., glycolysis, the tricarboxylic acid cycle, and the electron transport chain). In NIL-cml SAMs, most of the proteins with functions related to photosynthesis regulation and energy metabolism decreased markedly, whereas the amounts of most of the carbohydrate-metabolism-related proteins were increased compared to control (Supplementary Table S3). A general symptom of photosynthetic plants is energy deficit under non-inductive condition^35^,^36^. Zhang *et al*.^29^. found that *Agapanthus* plants showed significantly decreased photosynthesis rate under stress, which in turn resulted in energy deprivation. The energy shortage often leads to the enhancement of inherent pathways of carbohydrate metabolism to maintain energy for key metabolic processes^35^,^37^. Similarly, in this study, LD condition disrupts energy metabolism and photosynthesis and further enhances carbohydrate synthesis in NIL-cml juvenile vegetative phase. Based on previous published observations and our resent study results, we believe that ZmCCT has a negative role in LD-induced energy metabolism for flowering transition in maize.

### Proteins involved in RNA-, DNA-, and protein-binding factors

The expression of *ZmCCT* mRNA is mediated by the 5′-untranslated regions (UTRs) and the promoter region. Based on their putative molecular functions, 86 differentially expressed proteins related to nucleotide binding (RNA-, DNA-, and protein-binding factors) were identified in NIL-cml and H4 (Supplementary Table S2), and these binding factors constitute the main mechanism controlling protein activity in complex biological processes, such as developmental regulation and light-regulated morphogenesis^38^,^39^. In addition, many known transcription factors related to flowering regulation were also differentially expressed in our study, including ring zinc finger domain protein and nuclear transcription factor.

Our proteomic data showed that the level of the glycine-rich RNA-binding protein was increased in ZmCCT-positive maize NIL (NIL-cml-6/NIL-cml-3) from the vegetative to the induced stage, suggesting that the glycine-rich RNA-binding protein may regulate ZmCCT in NIL-cml plants. This pattern is consistent with that of calmodulin-binding protein (NIL-cml-3/H4-3 and NIL-cml-6/H4-6). The *Arabidopsis thaliana* glycine-rich RNA-binding proteins have an RNA recognition motif in the N-terminal domain and a glycine-rich C-terminal domain and are involved in a multitude of functions^40^. Glycine-rich RNA-binding proteins 7 and 8 show robust circadian oscillations of protein levels, forming the negative arm and promoting flowering by suppressing the expression of the FLC protein under noninductive conditions^41^,^42^. The *Arabidopsis thaliana* cytosolic calcium (Ca^2+^) levels also oscillate with a 24-h rhythm in plants. *Calmodulin (CaM)-like 24* (CML24) inhibits *FLC* expression and therefore influences flowering time, in conjunction with CML23^43^,^44^. Therefore, these results indicated that the proteins involved in RNA-, DNA-, or protein-binding may be connected with ZmCCT and regulate the maize flowering time in response to LD condition.

### Transcriptional expression patterns of six differentially expressed proteins

**Figure 6:**
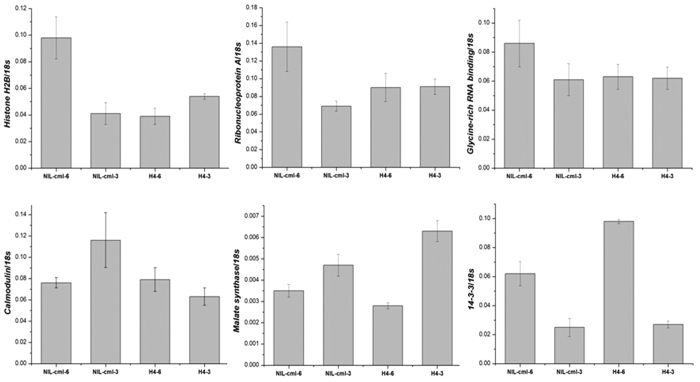
Real-time PCR quantitative analysis of six differentially expressed genes during vegetative to reproductive transition in NIL-cml and H4 plants. The average values (mean ± SEM) are based on three independent experiments.

In order to evaluate the correlation between mRNA and protein levels, we investigated the expression pattern of six representative genes (histone H2B, ribonucleoprotein A, glycine-rich RNA-binding protein, calmodulin-binding protein, malate synthase, and 14-3-3 protein) by quantifying the relative abundance of the respective mRNAs in the SAMs of maize using qPCR (Fig. 6). Under LD conditions in NIL SAMs, the expression levels of all these genes except malate synthase and 14-3-3 protein matched well with the iTRAQ data, suggesting that the abundance of these proteins is regulated at the transcriptional level. Although the mRNA expression of the gene encoding malate synthase is already down regulated from the V3 to the V6 stage, the protein levels continue to increase in NIL-cml plants. In contrast, the 14-3-3 protein showed a significant increase in mRNA levels but no significant variation in protein levels. Consistent with our results, several previous reports have revealed that enrichments of the identified proteins are probably regulated at the mRNA level, while others are regulated post-translationally^45^,^46^,^47^. The utility of these differentially expressed proteins should be further investigated in future studies.

### Protein–protein interaction analysis

**Figure 7:**
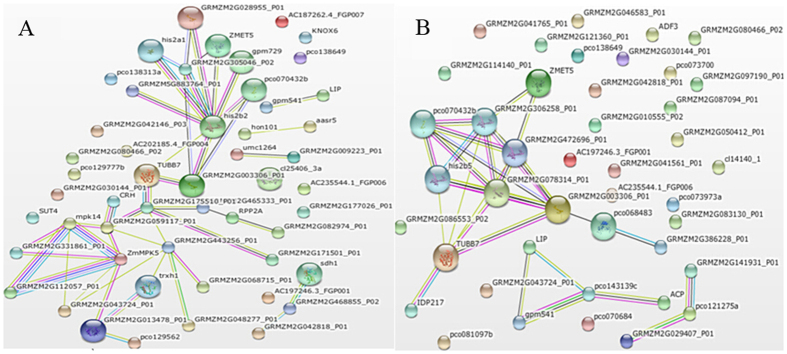
Protein-protein interaction network analyzed by STRING software. Network analysis results for significantly changed proteins (**A**) in sample group NIL-cml-3/H4-3 and (**B**) in sample group NIL-cml-6/H4-6. Different colors of the lines represent the types of evidence for association: green line, neighborhood evidence; red line, fusion evidence; purple line, experimental evidence; light blue line, database evidence; black line, coexpression evidence; blue line, co-occurrence evidence; and yellow line, text-mining evidence.

Most proteins exerted their biological functions by interacting with each other. To uncover functional aspects associated with these proteins, 52 significantly up- or down-regulated proteins were analyzed by searching the Search Tool for the Retrieval of Interacting Genes/Proteins (STRING) database (Fig. 7). These proteins were directly or indirectly induced by ZmCCT, which were associated mainly with carbohydrate metabolism, signal transduction, defense response, energy, photosynthesis, signal transduction, and unknown function (Supplementary Table S4; Fig. 7).

The protein interactions included the following: GRMZM2G112057-mpk14-ZmMPK5-GRMZM2G2913478-trxh1-GRMZM2G443256; GRMZM2G059117-TUBB7-GRMZM2G003306-his2b2- ZMET5; IDP217-GRMZM2G003306-GRMZM2G472696-ZMET5- GRMZM2G306258-pc0070432b-his2b5, and LIP-gpm541-pco143139c-ACP. For example, we found that the DNA (cytosine-5)-methyltransferase (ZMET5) and the leucine-rich repeat protein kinase (GRMZM2G059117) were detected in NIL-cml-6/H4-6 and NIL-cml-3/H4-3 (Supplementary Table S3). The INRNTPSADB motif was identified in the *ZmCCT* promoter, which exhibited cytosine methylation, and in turn methylation of the INRNTPSADB motif suppresses *ZmCCT* transcription in the temperate inbred line^12^, DNA (cytosine-5)-methyltransferase may participate in this process^48^. The leucine-rich repeat protein kinase was up-regulated in H4. In *Arabidopsis*, the leucine-rich repeat protein kinase play crucial roles in a variety of different physiological processes, including plant growth, development, pathogen resistance, and cell death^49^,^50^. Protein-protein interaction analysis showed that ZmCCT not only regulates the expression of the photoperiod pathway genes, but it affects genes in other pathways as well. The interaction between these proteins may have important roles on maize growth and development.

## Conclusions

*ZmCCT* is one of the most important genes affecting photoperiod response. Understanding the molecular and biochemical mechanisms of the role of ZmCCT is a vital step for integrating tropical germplasm into temperate zone maize breeding. In this work, morphological observations and proteomics approaches were applied to two maize lines (ZmCCT-positive/negative). Presence of ZmCCT resulted in stunting flowering transition in maize seedlings, which manifested in increased plant height and delay in flowering. Proteomic analysis indicated the abundance of 386 proteins that were differentially expressed between the ZmCCT-negative and positive plants. Functional categorization showed that the identified proteins were mainly involved in energy production, photosynthesis, signal transduction, and cell organization and biogenesis. Most of the proteins identified were located in membranes, cytoplasm, and nucleus.

In this work, both cytoskeletal proteins and histone proteins were more abundant in ZmCCT-positive plants, suggesting that ZmCCT might enhance cell division and growth during SAM development. Furthermore, the levels of proteins participating in photosynthesis and energy metabolism were down-regulated in NIL-cml SAMs, whereas carbohydrate metabolism-related proteins were increased compared to control, indicating that ZmCCT has a negative role in LD-induced energy metabolism for flowering transition in maize. In addition, there were a great number of binding proteins related to flowering regulation, which were also differentially expressed in our study. These proteins may directly or indirectly regulate the expression of the *ZmCCT* gene.

To date, this is the first study on the proteomic analysis of ZmCCT-regulated growth in maize, which will contribute to a better understanding of the molecular mechanism of photoperiodicity.

## Materials and Methods

### Plant materials and sample preparation

The inbred line Huangzao 4 (H4) and its NIL-cml were used to analyze the ZmCCT-associated protein. The NIL-cml was derived from the cross between Huangzao 4 (recurrent parent) and CML288 (nonrecurrent parent)^12^. CML288 is a tropical maize inbred line and highly sensitive to photoperiod, obtained from the National Maize and Wheat Improvement Center, Mexico, while Huangzao 4 is one of the basic genotypes that comprise the foundation of maize cross-breeding in China and which is insensitive to photoperiod. The plants were sown in 15 cm pots (four plants per pot) under LD conditions (15 h light and 9 h dark). For proteomic analysis, the SAMs were collected. Each sample was collected from six different, randomly selected plants. Three biological replicates were collected at the same time. All the materials were frozen in liquid nitrogen quickly after they were taken from the plant and then stored at –80 °C until use.

### Observations of SAM

In brief, the SAMs were first fixed with FAA, then rinsed several minutes in 70% ethanol, and peeled off under anatomical lens. The SAMs were then stained with 20 μg/ml Hoechst33258 (TaKaRa Biotechnology Company, Dalian, China) at 25 °C for 24 h in the dark. Finally, the morphology of maize SAM was observed under Leica TCS-SP2 laser scanning confocal microscope (Leica Technologies, Wetzlar, Germany).

### Protein Digestion and iTRAQ Labeling

Protein digestion was performed according to the filter-aided sample preparation (FASP) procedure described by Wiśniewski *et al*.^20^, and the resulting peptide mixture was labeled using the 4-plex/8-plex iTRAQ reagent according to the manufacturer’s instructions (Applied Biosystems). Briefly, 200 μg of proteins from each sample were incorporated into 30 μl STD buffer (4% sodium dodecyl sulfate, 100 mM dithiothreitol [DTT], 150 mM Tris-HCl pH 8.0). The detergent, DTT, and other low-molecular-weight components were removed using UA buffer (8 M urea, 150 mM Tris-HCl pH 8.0) by repeated ultrafiltration (Microcon units, 30 kDa). Then, 100 μl 0.05 M iodoacetamide in UA buffer was added to block the reduced cysteine residues, and the samples were incubated for 20 min in darkness. The filters were washed with 100 μl UA buffer three times and then with 100 μl DS buffer (50 mM triethylammonium bicarbonate at pH 8.5) twice. Finally, the protein suspensions were digested with 2 μg trypsin (Promega) in 40 μl DS buffer overnight at 37 °C, and the resulting peptides were collected as a filtrate. The peptide content was estimated by the UV light spectral density at 280 nm using an extinction coefficient of 1.1 for a 0.1% (g/l) solution, which was calculated on the basis of the frequency of tryptophan and tyrosine in vertebrate proteins.

For labeling, each iTRAQ reagent was dissolved in 70 μl of ethanol and added to the respective peptide mixture. The samples were labeled as (NIL-cml-6)-117, (NIL-cml-3)-118, (H4-6)-119, and (H4-3)-121 and were multiplexed and vacuum dried.

### Peptide Fractionation with Strong Cation Exchange Chromatography

iTRAQ-labeled peptides were fractionated by strong cation exchange (SCX) chromatography using the AKTA Purifier system (GE Healthcare). The dried peptide mixture was reconstituted and acidified with 2 ml buffer A (10 mM KH_2_PO_4_ in 25% of ACN, pH 2.7) and loaded onto a PolySULFOETHYL™ 4.6 × 100 mm column (5 μm, 200 Å, PolyLC Inc., Maryland, USA). The peptides were eluted at a flow rate of 1 ml/min with gradients of 0–10% buffer B (500 mM KCl, 10 mM KH_2_PO_4_ in 25% of ACN, pH 2.7) for 2 min, 10–20% buffer B for 25 min, 20–45% buffer B for 5 min, and 50–100% buffer B for 5 min. The elution was monitored by absorbance at 214 nm, and fractions were collected every 1 min. The collected fractions (about 30 fractions) were finally combined into 10 pools and desalted on C18 Cartridges (Empore™ SPE Cartridges C18 [standard density], bed inner diameter 7 mm, volume 3 ml, Sigma). Each fraction was concentrated by vacuum centrifugation and reconstituted in 40 μl of 0.1% (v/v) trifluoroacetic acid. All samples were stored at −80 °C until liquid chromatography-tandem mass spectrometry (LC-MS/MS) analysis.

### LC–Electrospray Ionization–MS/MS Analysis

Experiments were performed on a Q Exactive™ mass spectrometer that was coupled to Easy nLC (Proxeon Biosystems, now Thermo Fisher Scientific). Next, 10 μl of each fraction was injected for nano-LC-MS/MS analysis. The peptide mixture (5 μg) was loaded onto a C18 reversed-phase column (15 cm long, 75 μm inner diameter) packed in-house with RP-C18 5 μm resin in buffer A (0.1% formic acid) and separated with a linear gradient of buffer B (80% acetonitrile and 0.1% formic acid) at a flow rate of 250 nl/min, controlled by IntelliFlow technology, over a period of 140 min. MS data was acquired using a data-dependent top10 method, dynamically choosing the most abundant precursor ions from the survey scan (300–1,800 *m/z*) for high-energy collisional dissociation (HCD) fragmentation. Determination of the target value is based on predictive Automatic Gain Control. Dynamic exclusion duration was 60 s. Survey scans were acquired at a resolution of 70,000 at *m/z* 200 and resolution for HCD spectra was set to 17,500 at *m/z* 200. Normalized collision energy was 30 eV and the underfill ratio, which specifies the minimum percentage of the target value likely to be reached at maximum fill time, was defined as 0.1%. The instrument was run with peptide recognition mode enabled.

### Sequence Database Searching and Data Analysis

MS/MS spectra were searched using the MASCOT engine (Matrix Science, London, UK; version 2.2) embedded into Proteome Discoverer 1.3 (Thermo Electron, San Jose, CA, USA) against UniProt plant database (134,648 sequences) and the decoy database. For protein identification, the following options were used. Peptide mass tolerance = 20 ppm, MS/MS tolerance = 0.1 Da, enzyme = trypsin, missed cleavage = 2, fixed modification: carbamidomethyl (C), iTRAQ 4-plex (K), iTRAQ 4-plex (N-term), variable modification: oxidation (M), FDR ≤ 0.01. Only spectra in which all the expected iTRAQ reporter ions were detected were used for quantification. The protein ratios were normalized by dividing by the average value of all peptides identified. Student’s T test was used to evaluate the statistical significance, and the false discovery rate (Benjamini-Hochberg) was calculated to correct for multiple comparisons. To state that a protern has a significant abundance changes between two samples, the following criteria have to be fulfilled: the abundance ratios has to be ≥1.5 and the P-value for student’s t test has to be less than 0.05^23^,^24^.

### Bioinformatics

Molecular functions of the identified phosphoproteins were classified according to their gene ontology (GO) annotations combined with their biological function. Subcellular locations of unique phosphoproteins identified in this study were determined from the UniProt database (http://www.uniprot.org) or predicted using the publicly available program, WoLF PSORT (http://wolfpsort.org).

### Western blot analysis

For each of the four maize samples, the extracted total proteins (15 μg) were separated on 12% SDS PAGE gels and then transferred onto a polyvinylidene difluoride (PVDF) membrane using an electrophoretic transfer system (Bio-Rad, USA). Membranes were blocked for 1 h at room temperature with 5% skim milk in PBST and probed with rabbit polyclonal antibody to ascorbate peroxidase (Agrisera, Sweden), rabbit polyclonal antibody to peroxiredoxin (Agrisera, Sweden), rabbit polyclonal antibody to superoxide dismutase (Agrisera, Sweden), and mouse monoclonal antibody to actin (Abmart, China) at 4 °C overnight respectively. Then the membranes were incubated with horseradish peroxidase (HRP) conjugated goat antirabbit IgG or goat antimouse IgG (Boshide, China) for 1 h at room temperature. Immunoreactivity was detected with an HRP-DAB Detection Kit (Tiangen, China).

### Quantitative real-time polymerase chain reaction analysis

Roots, leaves, SAMs, ligules and sheath were collected at the same time for protein and total RNA isolation. Total RNA was isolated from each tissue sample using TRIZOL reagent (Invitrogen, Carlsbad, CA, USA) according to the manufacturer’s instructions. Each RNA sample was treated with RNase-free DNase (TaKaRa, Dalian, China). Complementary DNA was reverse-transcribed from 2 μg of total RNA using PrimeScript™ 1st Strand cDNA Synthesis Kit (TaKaRa) following the manufacturer’s instructions. Quantitative real-time polymerase chain reaction (qPCR) was performed on a Bio-Rad real-time detection system (Bio-Rad, Hercules, CA, USA). The reaction volume was 25 μl, consisting of 10 × PCR buffer (Mg^2+^), 4 μl; 10 mmol L^–1^ dNTP, 0.5 μl; Plus-Taq, 0.3 μl; 20 × SYBR dye, 1 μl; gene-specific primers (forward; reverse), 0.6 μl for a final 20 pmol/L concentration; water, 16 μl and cDNA template, 2 μl. Each reaction was performed in triplicate.

qPCR experiments were conducted following MIQE guidelines^51^, the constitutive gene *18S* (GenBank accession no. AF168884.1) was used as endogenous control to normalize expression in maize SAMs^52^. The quantification of gene expression levels was calculated relative to *18S* using a 2^–ΔΔCT^ method^53^. The primers for the qPCR assay for each candidate gene and *18S* were designed using the Primer5.0 software (PREMIER Biosoft International, CA, USA) and are listed in Supplementary Table S1.

## Additional Information

**How to cite this article**: Wu, L. *et al*. Comparative proteomic analysis of the shoot apical meristem in maize between a ZmCCT-associated near-isogenic line and its recurrent parent. *Sci. Rep.* **6**, 30641; doi: 10.1038/srep30641 (2016).

## Supplementary Material

Supplementary Figures

Supplementary Tables

Supplementary Dataset S2

Supplementary Dataset S3

## Figures and Tables

**Table 1:** Phenotypes of two maize near-isogenic lines (n = 10) under long day conditions.

Materials	Plant height (cm)	Days to pollen shedding	Days to silk
H4	127.8 (0.39)	64.3 (0.32)	69.5 (0.22)
NIL-cml	164.3 (0.45)	71.5 (0.26)	79.2 (0.43)

Standard errors of means are shown in parentheses.
