# Supplementary material for: Comparative proteomic analysis of the shoot apical meristem in maize between a ZmCCT-associated near-isogenic line and its recurrent parent
Source: Sci Rep. 2016 Jul 29;6:30641. doi: 10.1038/srep30641 (PMC4965789; doi:10.1038/srep30641)

Comparative proteomic analysis of the shoot apical meristem in maize between a ZmCCT-associated near-isogenic line and its recurrent parent

Liuji Wu[1,2†], Xintao Wang[3†], Shunxi Wang[1,2], Liancheng Wu[1,2], Lei Tian[1, 2], Zhiqiang Tian[1,2], Ping Liu[1, 2], and Yanhui Chen[1,2]*

Legends for Supplemental Figures

Figure S1 Changes in SAM height (μm) from the NIL-cml and H4 lines (n=10) under LD conditions. Error bars indicate SD (n=10).

Figure S2 Relative abundance of the *ZmCCT* transcripts in SAMs from the V3 to the V10 stage (A) and in different maize tissues (B). The average values (mean ± SEM) are based on three independent experiments.

Figure S3 iTRAQ 4-plex Labeling and LC MS/MS workflow of identifying phosphoros proteins in maize SAMs of H4 and its NIL-cml seedlings under long day (LD) conditions.

Figure S4 Comparative analysis of differentially expressed proteins among three replicates in V3 and V6 stage of NIL-cml and H4. Venn diagram showing the distribution of common and exclusive proteins among three replicates in NIL-cml-3/H4-3 (A), NIL-cml-6/H4-6 (B), NIL-cml-6/NIL-cml-3 (C) and H4-6/H4-3 (D), respectively. The areas shown in the diagram are not proportional to the number of proteins in each group.

Figure S5 Volcano plot of the identified proteins with expression levels changes in V3 and V6 stage of NIL-cml and H4. A, B , C and D represent the identified proteins with expression levels in NIL-cml-3/H4-3, NIL-cml-6/H4-6, NIL-cml-6/NIL-cml-3 and H4-6/H4-3, respectively. Volcano plot distributions of fold change (log2 [fold change]) (X-axis) and Student's t-test p-values (-log10 [p-value]) (Y-axis). The −log10 (Benjamin–Hochberg corrected P value) is plotted against the log2 (fold

change). The non-axial vertical lines denote ±1.5-fold change while the non-axial horizontal line denotes P = 0.05, which is our significance threshold (prior to logarithmic transformation).

Figure S1

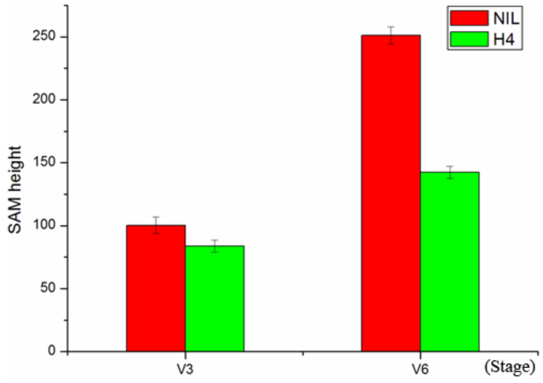

Figure S2

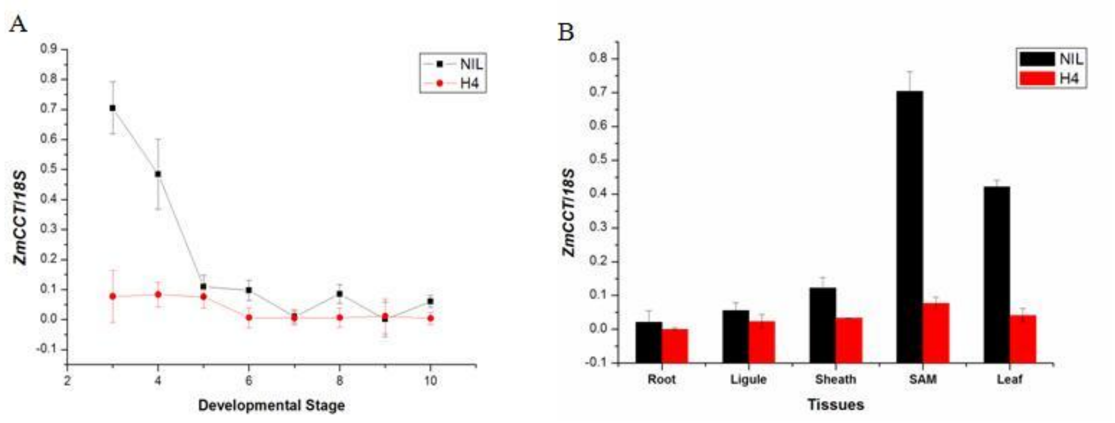

Figure S3

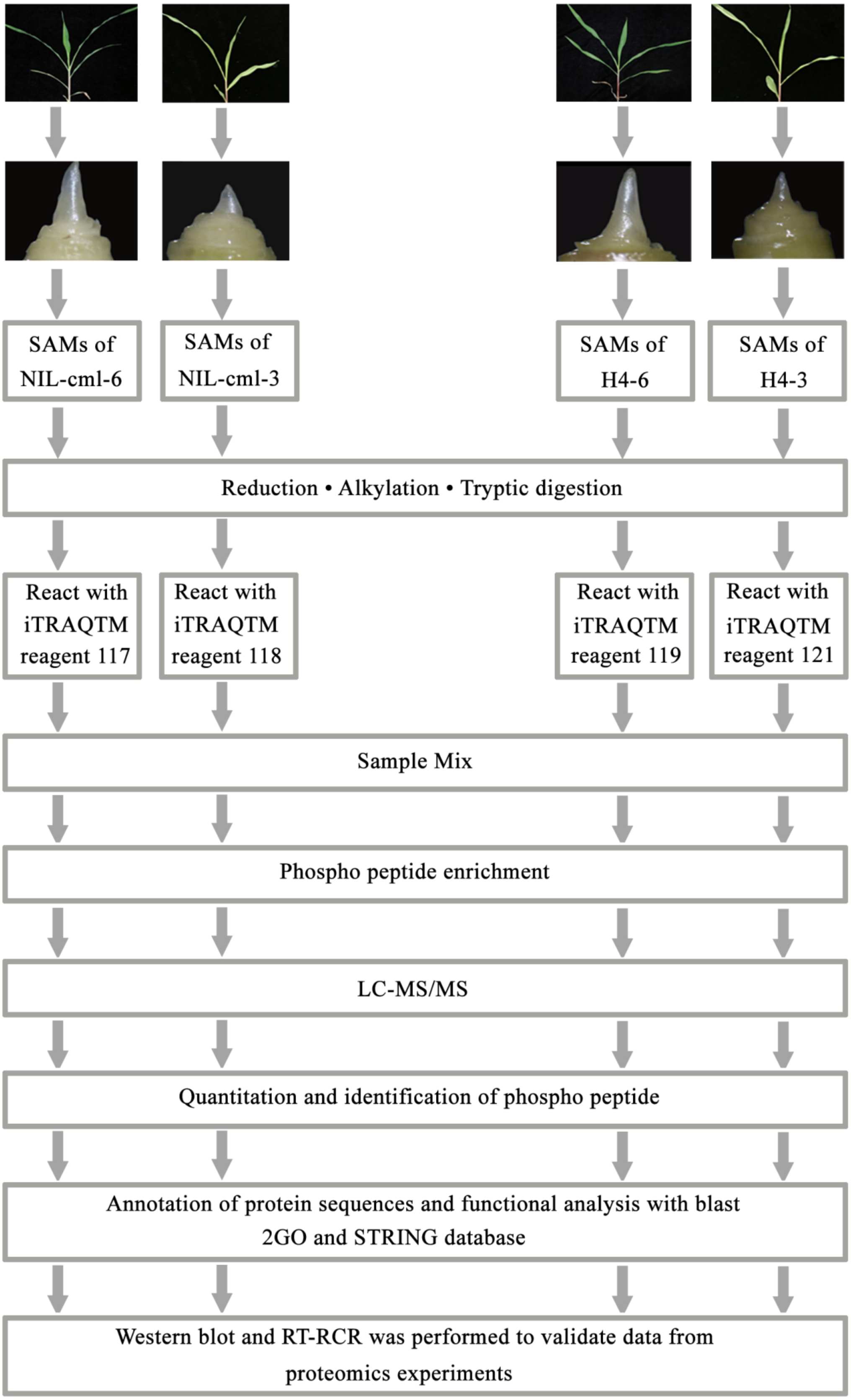
SAMs of NIL-cml-6
SAMs of NIL-cml-3
SAMs of H4-6
SAMs of H4-3
Reduction • Alkylation • Tryptic digestion
React with iTRAQTM reagent 117
React with iTRAQTM reagent 118
React with iTRAQTM reagent 119
React with iTRAQTM reagent 121
Sample Mix
Phospho peptide enrichment
LC-MS/MS
Quantitation and identification of phospho peptide
Annotation of protein sequences and functional analysis with blast 2GO and STRING database
Western blot and RT-RCR was performed to validate data from proteomics experiments

Figure S4

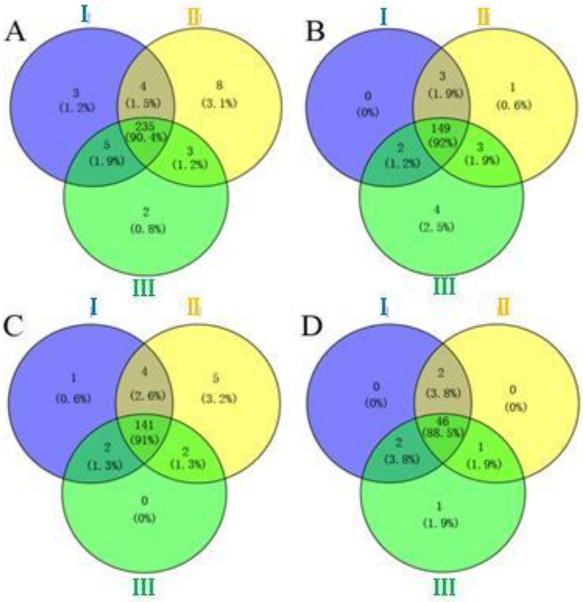

Figure S5

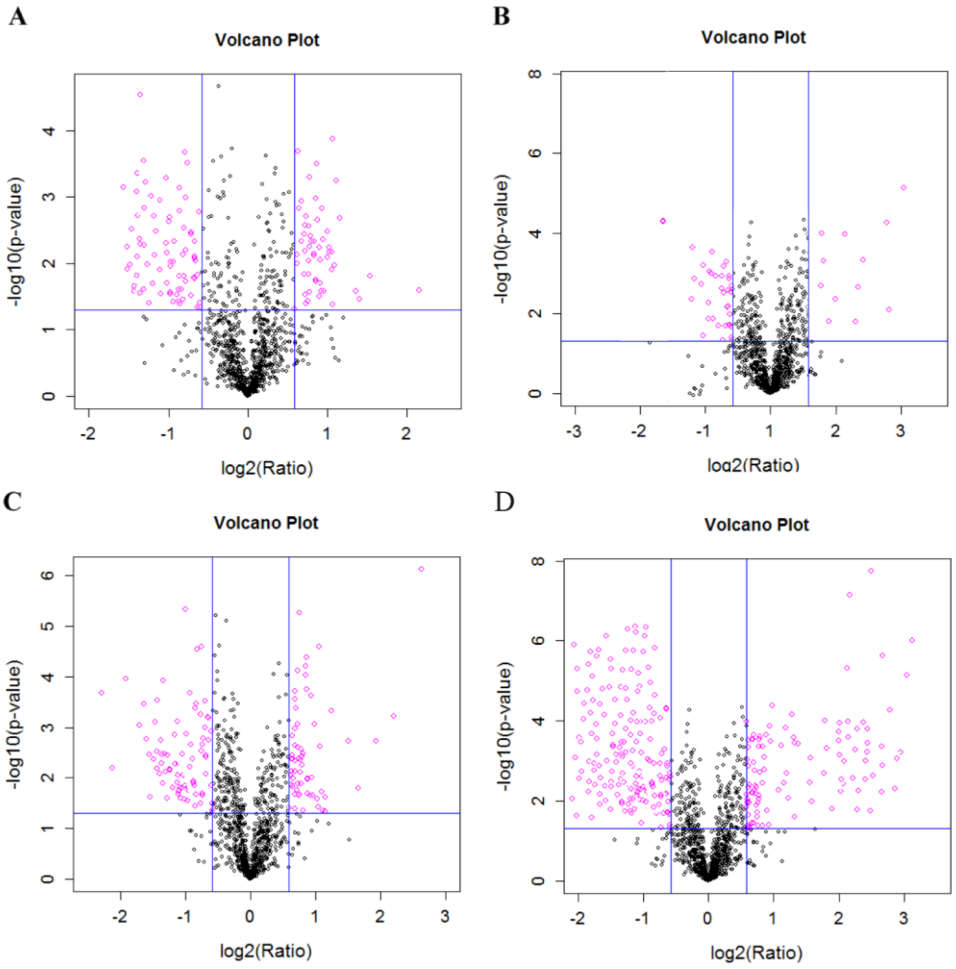

Supplement: Supplementary Figures [file srep30641-s1.pdf]
