# Supplementary material for: Comparative proteomic analysis of the shoot apical meristem in maize between a ZmCCT-associated near-isogenic line and its recurrent parent
Source: Sci Rep. 2016 Jul 29;6:30641. doi: 10.1038/srep30641 (PMC4965789; doi:10.1038/srep30641)
Supplement: Supplementary Tables [file srep30641-s2.pdf]

Comparative proteomic analysis of the shoot apical meristem in maize between a ZmCCT-associated near-isogenic line and its recurrent parent

Liuji Wu[1,2†], Xintao Wang[3†], Shunxi Wang[1,2], Liancheng Wu[1,2], Lei Tian[1, 2], Zhiqiang Tian[1,2], Ping Liu[1, 2], and Yanhui Chen[1,2]*

Table S1. Primer sets for real-time PCR analysis in this study.

Target genes	Primers (5'→3')	Amplification efficiency (%)	R^2
Histone H2B	F: 5' AGGCAAAGAAGTCGGTGGA 3'	98.6	0.996
	R: 5' GCGAACGGAGGTCTGGAT 3'		
Ribonucleoprotein A	F: 5' AAGGAAGACAACTGAGGGTGA 3'	99.2	0.998
	R: 5' GGAGCCATAGGTGACGAAA 3'		
Glycine-rich RNA binding protein	F: 5' GCTTCGGCTTCGTCACCTTCTC 3'	101.3	0.998
	R: 5' TCCTCCAGTTCCCGTCGTTGTT 3'		
Calmodulin binding protein isoform 1	F: 5' ACGCCACGCTCCGCTGCAT 3'	98.4	0.997
	R: 5' TCGCCCACGTCCACCTCCAC 3'		
Malate synthase, glyoxysomal	F: 5' GCCTCTACTTCTTCCACAACCACG 3'	99.6	0.996
	R: 5' GGCTCGATCCCAGCAGCTTTCT 3'		
14-3-3	F: 5' GGCTAAGACCGTAGATGTG 3'	97.5	0.997
	R: 5' CGATGGAGGAGATAATGC 3'		
ZmCCT	F: 5' TCCGTCTTCCCTGTCGT 3'	99.3	0.999
	R: 5' CCAGGCGATGGTTTCTT 3'		
18S	F: 5' CCTGCGGCTTAATTGACTC 3'	100.6	0.997
	R: 5' GTTAGCAGGCTGAGGTCTCG 3'		

Table S4. The abbreviations of the specific protein names in the protein-protein interaction networks.

4.1 NIL-cml-3 / H4-3

Abbreviation	Protein name
AC187262.4_FGP007	hypothetical protein
AC197246.3_FGP001	ras-related protein ARA-4
AC202185.4_FGP004	hsp20/alpha crystallin family protein
AC235544.1_FGP006	Legumin-like protein
GRMZM2G003306_P01	Histone H2A
ZMET5	DNA (cytosine-5)-methyltransferase 3
GRMZM2G009223_P01	glucose-6-phosphate/phosphate translocator 2
pco138313a	hypothetical protein
GRMZM2G013478_P01	nucleoside diphosphate kinase 2
pco138649	hypothetical protein
ZmMPK5	MAP kinase 5
umc1264	LOC100282085
GRMZM2G028955_P01	hypothetical protein
GRMZM2G030144_P01	hypothetical protein
pco129562	hypothetical protein
GRMZM2G042146_P03	Putative uncharacterized protein
GRMZM2G042818_P01	3-hydroxyisobutyryl-CoA hydrolase/ catalytic

GRMZM2G043724_P01	ATP-dependent RNA helicase DDX41
TUBB7	Tubulin beta-7 chain (Beta-7-tubulin)
GRMZM2G048277_P01	peptidyl-prolyl cis-trans isomerase NIMA-interacting 4
aasr5	Asr protein
GRMZM2G059117_P01	uncharacterized LOC100384143
pco129777b	LOC100282545
mpk14	MPK14 - putative MAPK
GRMZM2G068715_P01	ribonucleoprotein A
hon101	histone one (H1) 101
GRMZM2G080466_P02	LOC100284786
GRMZM2G082974_P01	hypothetical protein LOC100192991
cl25406_3a	LOC100286223
LIP	Malate synthase, glyoxysomal
GRMZM2G112057_P01	calcium-dependent protein kinase
his2b2	Histone H2B.2
RPP2A	60S acidic ribosomal protein P2A (P2)
gpm729	hypothetical protein
GRMZM2G141931_P01	endoplasmin
pco070432b	hypothetical protein
GRMZM2G171501_P01	50S ribosomal protein L12-2
sdh1	sorbitol dehydrogenase homolog1
GRMZM2G175510_P01	Putative uncharacterized protein
GRMZM2G177026_P01	hypothetical protein
GRMZM2G305046_P02	Histone H2A
SUT4	sucrose transporter4 (501 aa)
GRMZM2G331861_P01	Putative pyridoxal phosphate (PLP)-dependent transferase family protein
CRH	Calreticulin Precursor
his2a1	Histone H2A
KNOX6	Homeobox protein knotted-1-like 7 Fragment
trxh1	thioredoxin h1 protein
gpm541	hypothetical protein
GRMZM2G443256_P01	SMAD/FHA domain-containing family protein
GRMZM2G465333_P01	nascent polypeptide-associated complex alpha subunit-like protein
GRMZM2G468855_P02	hypothetical protein LOC100273052
GRMZM5G883764_P01	Histone H2A

4.2 NIL-cml-6 / H4-6

Abbreviation	Protein name
AC197246.3_FGP001	ras-related protein ARA-4
AC235544.1_FGP006	Legumin-like protein
GRMZM2G003306_P01	Histone H2A
pco121275a	LOC100283671
ZMET5	DNA (cytosine-5)-methyltransferase 3
GRMZM2G010555_P02	immutans protein
pco138649	hypothetical protein
pco073973a	LOC100283624
GRMZM2G029407_P01	hypothetical protein
GRMZM2G030144_P01	hypothetical protein
pco070684	LOC100284619
GRMZM2G041561_P01	BRCA1-associated protein
GRMZM2G041765_P01	IQ calmodulin-binding motif family protein

GRMZM2G042818_P01	3-hydroxyisobutyryl-CoA hydrolase/ catalytic
GRMZM2G043724_P01	ATP-dependent RNA helicase DDX41
TUBB7	Tubulin beta-7 chain (Beta-7-tubulin)
pco081097b	LOC100284848
GRMZM2G046583_P01	nicotinate phosphoribosyltransferase-like protein
pco073700	LOC100283824
GRMZM2G050412_P01	hypothetical protein LOC100274518
ADF3	Actin-depolymerizing factor 3(ZmADF3)
cl14140_1	LOC100282110
GRMZM2G078314_P01	Histone H3
GRMZM2G080466_P02	LOC100284786
GRMZM2G083130_P01	cytokinin-O-glucosyltransferase 2
GRMZM2G086553_P02	AT-hook protein 1
GRMZM2G087094_P01	VAMP protein SEC22
ACP	Acyl carrier protein
GRMZM2G097190_P01	nucleotide-binding protein 1
LIP	Malate synthase, glyoxysomal
GRMZM2G114140_P01	hydrolase, hydrolyzing O-glycosyl compounds
GRMZM2G121360_P01	hypothetical protein
pco068483	hypothetical protein
GRMZM2G141931_P01	endoplasmin
IDP217	LOC100281977
pco070432b	hypothetical protein
pco143139c	hypothetical protein
GRMZM2G306258_P01	Histone H2B.4
his2b5	Histone H2B.5 (H2B)
GRMZM2G386228_P01	protein transport protein Sec61 beta subunit
gpm541	hypothetical protein
GRMZM2G472696_P01	Histone H2B
